# 

**Published:** 1908-05-30

**Authors:** 


### CONTENTS.

| | PAGE |
|---|---|
| **The Endowment of Pathological Research** | 217 |
| **Overlay or Overlie** | 218 |
| **Annotations—** | |
| Tuberculous Meat—International Cancer Research—Medical Reciprocity with Canada | 219 |
| **Medical Opinion and Movement** | 220 |
| **Hospital Clinics—** | |
| Syphilitic Cachexia. By Sir Dyce Duckworth, M.D., LL.D., F.R.C.P. | 223 |
| **Medicine—** | |
| Phthisis with Perforation of the Bowel | 226 |
| Serous Hæmorrhage | 228 |
| **Points in Surgery—** | |
| Some Aspects of Intestinal Obstruction | 229 |
| Saline Infusion | 230 |
| **Practical Notes on Diagnosis and Treatment** | 230 |
| **Laryngology and Rhinology—** | |
| Nasal Polypi | 231 |
| **The General Practitioner's Column—** | |
| An Interesting Case of Cerebral Tumour | 232 |
| **Dental Department—** | |
| The Present Condition of the Dental Profession | 233 |
| **Therapeutics—** | |
| Secret Remedies and Branded Products—VI. | 235 |
| Ammonia in Bismuth Subnitrate | 236 |
| **Cases for Diagnosis—** | |
| Two Cases of Acute Abdominal Disease | 237 |
| **New Appliances and Things Medical** | 238 |
| **Hospital Administration—** | |
| Graduate Study on the Continent—Germany | 239 |
| Social and Poor-law Problems | 240 |
| News and Coming Events | 241 |
| **Nursing Administration—** | |
| The Bedding and Linen Department—V. | 243 |
| **Editor's Letter-Box** | 244 |
| **The Graduates' Medical Schools** | 244 |

